# *cyclo*-Hexa-μ_2_-diphenyl­acetato-κ^12^ *O*:*O*′-hexa-μ_3_-oxido-hexa­kis[phenyl­tin(IV)]

**DOI:** 10.1107/S1600536808038567

**Published:** 2008-11-26

**Authors:** Mostafa M. Amini, Taraneh Hajiashrafi, Ali Nemati Kharat, Seik Weng Ng

**Affiliations:** a Department of Chemistry, Shahid Beheshti University, Tehran, Iran; b School of Chemistry, College of Science, Tehran University, Tehran, Iran; c Department of Chemistry, University of Malaya, 50603 Kuala Lumpur, Malaysia

## Abstract

In the cyclo­hexa­meric centrosymmetric title compound, [Sn_6_(C_6_H_5_)_6_(C_14_H_11_O_2_)_6_O_6_], each bridging oxide O atom is connected to three Sn atoms and each carboxyl­ate group bridges a pair of Sn atoms so that the three independent Sn atoms all adopt SnCO_5_ distorted octa­hedral geometries, resulting in a drum-shaped Sn_6_O_6_ core.

## Related literature

For examples of drum-shaped cyclo­hexa­meric oxophenyl­tin(IV) carboxyl­ates, see: Alcock & Roe (1989 ▶); Chandrasekhar *et al.* (1985 ▶); Yin *et al.* (2003 ▶); Zhang *et al.* (2005 ▶). For reviews of organotin carboxyl­ates, see: Tiekink (1991 ▶, 1994 ▶).
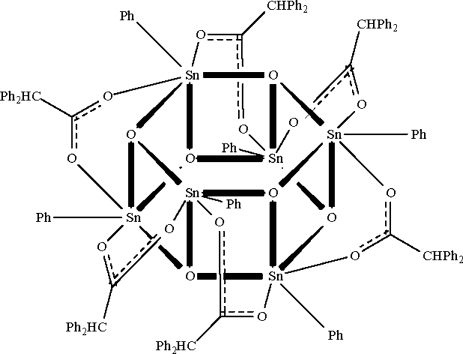


## Experimental

### 

#### Crystal data

[Sn_6_(C_6_H_5_)_6_(C_14_H_11_O_2_)_6_O_6_]
*M* *_r_* = 2538.11
Triclinic, 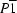

*a* = 12.9375 (2) Å
*b* = 14.7406 (2) Å
*c* = 14.8043 (2) Å
α = 103.368 (1)°
β = 111.716 (1)°
γ = 92.582 (1)°
*V* = 2525.06 (6) Å^3^
*Z* = 1
Mo *K*α radiation
μ = 1.53 mm^−1^
*T* = 112 (2) K
0.25 × 0.20 × 0.17 mm

#### Data collection

Bruker APEXII diffractometer
Absorption correction: multi-scan (*SADABS*; Sheldrick, 1996 ▶) *T*_min_ = 0.701, *T*_max_ = 0.781
37375 measured reflections
11415 independent reflections
9692 reflections with *I* > 2σ(*I*)
*R*_int_ = 0.041

#### Refinement

*R*[*F* ^2^ > 2σ(*F* ^2^)] = 0.034
*wR*(*F* ^2^) = 0.117
*S* = 1.18
11415 reflections
541 parameters
H-atom parameters constrained
Δρ_max_ = 0.94 e Å^−3^
Δρ_min_ = −2.31 e Å^−3^

### 

Data collection: *APEX2* (Bruker, 2004 ▶); cell refinement: *SAINT* (Bruker, 2004 ▶); data reduction: *SAINT*; program(s) used to solve structure: *SHELXS97* (Sheldrick, 2008 ▶); program(s) used to refine structure: *SHELXL97* (Sheldrick, 2008 ▶); molecular graphics: *X-SEED* (Barbour, 2001 ▶); software used to prepare material for publication: *publCIF* (Westrip, 2008 ▶).

## Supplementary Material

Crystal structure: contains datablocks global, I. DOI: 10.1107/S1600536808038567/hb2814sup1.cif

Structure factors: contains datablocks I. DOI: 10.1107/S1600536808038567/hb2814Isup2.hkl

Additional supplementary materials:  crystallographic information; 3D view; checkCIF report

## Figures and Tables

**Table 1:** Selected bond lengths (Å)

Sn1—O9^i^	2.070 (3)
Sn1—O8	2.082 (3)
Sn1—O7	2.094 (3)
Sn1—O3	2.153 (3)
Sn1—O1	2.170 (3)
Sn1—C1	2.1336 (18)
Sn2—O7	2.072 (3)
Sn2—O9	2.088 (3)
Sn2—O8	2.089 (3)
Sn2—O4	2.163 (3)
Sn2—O5	2.171 (3)
Sn2—C7	2.1250 (18)
Sn3—O7^i^	2.073 (3)
Sn3—O8	2.076 (3)
Sn3—O9	2.125 (3)
Sn3—O2^i^	2.148 (3)
Sn3—O6	2.158 (3)
Sn3—C13	2.1181 (18)

Symmetry code: (i) 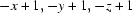.

**Fig. 1.**
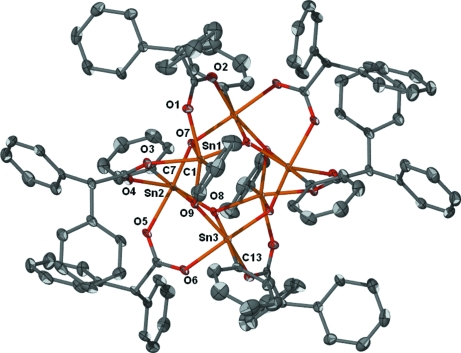
View of (I) with displacement ellipsoids for the non-hydrogen atoms drawn at the 70% probability level.

## supplementary crystallographic information

### Comment

The drum-shaped Sn_6_O_6_ core found for the title compound, (I), cyclohexameric [SnO(C_6_H_5_)[O_2_CCH(C_6_H_5_)_2_]_6_ or triphenyltin 2-hydroxy-3-phenylpropionate (Fig. 1) is similar to those seen in related structures (Alcock & Roe, 1989; Chandrasekhar *et al.*, 1985; Yin *et al.*, 2003; Zhang *et al.*, 2005. For reviews of organotin carboxylates, see: Tiekink (1991, 1994).

### Experimental

Triphenyltin hydroxide (1.0 g, 2.7 mmol) and diphenylacetic acid (0.58 g, 2.7 mmol) were heated in toluene (100 ml) in a Dean-Stark water separator until all the water had been removed. The solvent was removed under reduced pressure to leave a white powder; colourless blocks of (I) were recrystallized from a mixture of hexane and diethyl ether (1:1 *v*/*v*) at 268 K (m.p. 370–371 K).

IR (KBr, cm^-1^): 1551 (CO, *asym*), 1412 (CO, *sym*), 566, 609 (Sn—C). ^1^HNMR (CDCl_3_): 2.21 (s, 1H),6.7–7.7 (20*H*, C_6_H_5_) p.p.m.. ^13^CNMR (CDCl_3_): 59.7 (CH), 128.4(C*_meta_*), 129.0 (C*_para_*), 136.8 (C*_ortho_*), 137.8(C*_ipso_*), 180.0 (CO) p.p.m.. ^119^SnNMR (CDCl_3_): -85.6 p.p.m.. Mass spectrum (*m*/*e*): 485 (M—Ph) [Ph_3_SnO_2_CCHPh]^+^.

### Refinement

The aromatic rings were refined as rigid hexagons (C—C = 1.39 Å). The carbon-bound hydrogen atoms were placed in calculated positions (C—H = 0.95Å) and refined as riding with U_iso_(H) = 1.2U_eq_(C). The deepest difference hole is 1 Å from Sn2.

### Crystal data

**Table:**

[Sn_6_(C_6_H_5_)_6_(C_14_H_11_O_2_)_6_O_6_]	*Z* = 1
*M**_r_* = 2538.11	*F*_000_ = 1260
Triclinic, *P*1	*D*_x_ = 1.669 Mg m^−^^3^
Hall symbol: -P 1	Mo *K*α radiation λ = 0.71073 Å
*a* = 12.9375 (2) Å	Cell parameters from 9896 reflections
*b* = 14.7406 (2) Å	θ = 2.4–32.5º
*c* = 14.8043 (2) Å	µ = 1.53 mm^−^^1^
α = 103.368 (1)º	*T* = 112 (2) K
β = 111.716 (1)º	Block, colorless
γ = 92.582 (1)º	0.25 × 0.20 × 0.17 mm
*V* = 2525.06 (6) Å^3^	

### Data collection

**Table:**

Bruker APEXII diffractometer	11415 independent reflections
Radiation source: medium-focus sealed tube	9692 reflections with *I* > 2σ(*I*)
Monochromator: graphite	*R*_int_ = 0.041
*T* = 112(2) K	θ_max_ = 27.5º
φ and ω scans	θ_min_ = 1.4º
Absorption correction: Multi-scan(SADABS; Sheldrick, 1996)	*h* = −16→16
*T*_min_ = 0.701, *T*_max_ = 0.781	*k* = −19→19
37375 measured reflections	*l* = −19→19

### Refinement

**Table:**

Refinement on *F*^2^	Secondary atom site location: difference Fourier map
Least-squares matrix: full	Hydrogen site location: inferred from neighbouring sites
*R*[*F*^2^ > 2σ(*F*^2^)] = 0.034	H-atom parameters constrained
*wR*(*F*^2^) = 0.118	*w* = 1/[σ^2^(*F*_o_^2^) + (0.0656*P*)^2^ + 0.8272*P*] where *P* = (*F*_o_^2^ + 2*F*_c_^2^)/3
*S* = 1.18	(Δ/σ)_max_ = 0.001
11415 reflections	Δρ_max_ = 0.94 e Å^−^^3^
541 parameters	Δρ_min_ = −2.31 e Å^−^^3^
Primary atom site location: structure-invariant direct methods	Extinction correction: none

### Fractional atomic coordinates and isotropic or equivalent isotropic displacement parameters (Å^2^)

**Table:**

	*x*	*y*	*z*	*U*_iso_*/*U*_eq_	
Sn1	0.53479 (2)	0.665112 (17)	0.603202 (18)	0.00889 (7)	
Sn2	0.41572 (2)	0.479921 (17)	0.623742 (18)	0.00872 (7)	
Sn3	0.65401 (2)	0.436746 (17)	0.615334 (18)	0.00909 (7)	
O1	0.4513 (2)	0.78091 (19)	0.5569 (2)	0.0138 (6)	
O2	0.3127 (2)	0.70644 (19)	0.4092 (2)	0.0134 (6)	
O3	0.4792 (2)	0.71780 (18)	0.7232 (2)	0.0126 (5)	
O4	0.3990 (2)	0.58887 (18)	0.7415 (2)	0.0134 (6)	
O5	0.4928 (2)	0.42566 (19)	0.7539 (2)	0.0131 (5)	
O6	0.6614 (2)	0.40039 (19)	0.7504 (2)	0.0133 (6)	
O7	0.3820 (2)	0.57419 (18)	0.53567 (19)	0.0096 (5)	
O8	0.5761 (2)	0.54875 (18)	0.65948 (19)	0.0099 (5)	
O9	0.4812 (2)	0.37808 (18)	0.54526 (19)	0.0100 (5)	
C1	0.68854 (19)	0.75777 (16)	0.69348 (17)	0.0130 (8)	
C2	0.7322 (2)	0.82506 (19)	0.66001 (16)	0.0198 (9)	
H2	0.7016	0.8237	0.5906	0.024*	
C3	0.8206 (2)	0.89432 (17)	0.7281 (2)	0.0286 (11)	
H3	0.8504	0.9403	0.7053	0.034*	
C4	0.8653 (2)	0.89630 (19)	0.8297 (2)	0.0292 (11)	
H4	0.9257	0.9436	0.8763	0.035*	
C5	0.8216 (2)	0.8290 (2)	0.86321 (15)	0.0335 (12)	
H5	0.8522	0.8304	0.9326	0.040*	
C6	0.7333 (2)	0.75976 (19)	0.79508 (19)	0.0251 (10)	
H6	0.7034	0.7138	0.8180	0.030*	
C7	0.25612 (17)	0.40475 (17)	0.5900 (2)	0.0135 (8)	
C8	0.1648 (2)	0.45162 (14)	0.5880 (2)	0.0214 (9)	
H8	0.1717	0.5179	0.5972	0.026*	
C9	0.06332 (19)	0.4015 (2)	0.5725 (3)	0.0281 (11)	
H9	0.0009	0.4336	0.5711	0.034*	
C10	0.05322 (19)	0.3045 (2)	0.5590 (3)	0.0364 (13)	
H10	−0.0161	0.2703	0.5484	0.044*	
C11	0.1446 (2)	0.25768 (14)	0.5610 (3)	0.0339 (12)	
H11	0.1377	0.1914	0.5518	0.041*	
C12	0.2460 (2)	0.30777 (17)	0.5765 (2)	0.0229 (9)	
H12	0.3085	0.2757	0.5779	0.028*	
C13	0.82641 (15)	0.49468 (18)	0.68507 (19)	0.0124 (7)	
C14	0.86491 (19)	0.57261 (18)	0.66235 (19)	0.0182 (8)	
H14	0.8135	0.6012	0.6165	0.022*	
C15	0.9786 (2)	0.60878 (17)	0.7066 (2)	0.0259 (10)	
H15	1.0049	0.6620	0.6911	0.031*	
C16	1.05382 (16)	0.5670 (2)	0.7737 (2)	0.0276 (10)	
H16	1.1315	0.5917	0.8039	0.033*	
C17	1.01532 (19)	0.4891 (2)	0.7964 (2)	0.0262 (10)	
H17	1.0667	0.4605	0.8422	0.031*	
C18	0.9016 (2)	0.45292 (16)	0.7521 (2)	0.0191 (9)	
H18	0.8753	0.3997	0.7676	0.023*	
C19	0.3723 (3)	0.7794 (3)	0.4741 (3)	0.0126 (8)	
C20	0.3523 (3)	0.8741 (3)	0.4514 (3)	0.0135 (8)	
H20	0.3833	0.9233	0.5175	0.016*	
C21	0.22811 (16)	0.88231 (19)	0.40101 (19)	0.0155 (8)	
C22	0.1608 (2)	0.82467 (17)	0.30603 (19)	0.0199 (9)	
H22	0.1920	0.7789	0.2711	0.024*	
C23	0.0479 (2)	0.83396 (19)	0.26215 (17)	0.0267 (10)	
H23	0.0019	0.7946	0.1972	0.032*	
C24	0.00225 (16)	0.9009 (2)	0.3133 (2)	0.0271 (10)	
H24	−0.0749	0.9072	0.2833	0.033*	
C25	0.0695 (2)	0.95852 (19)	0.4082 (2)	0.0264 (10)	
H25	0.0384	1.0043	0.4432	0.032*	
C26	0.1825 (2)	0.94924 (18)	0.45212 (16)	0.0207 (9)	
H26	0.2285	0.9886	0.5170	0.025*	
C27	0.4212 (2)	0.89411 (18)	0.38980 (19)	0.0149 (8)	
C28	0.5037 (2)	0.84157 (16)	0.3770 (2)	0.0194 (9)	
H28	0.5175	0.7884	0.4035	0.023*	
C29	0.5661 (2)	0.86681 (19)	0.3254 (2)	0.0240 (9)	
H29	0.6225	0.8309	0.3167	0.029*	
C30	0.5459 (2)	0.94460 (19)	0.2867 (2)	0.0251 (10)	
H30	0.5885	0.9619	0.2514	0.030*	
C31	0.4634 (2)	0.99714 (16)	0.2995 (2)	0.0226 (9)	
H31	0.4496	1.0503	0.2730	0.027*	
C32	0.4010 (2)	0.97190 (17)	0.3511 (2)	0.0189 (9)	
H32	0.3446	1.0078	0.3598	0.023*	
C33	0.4280 (3)	0.6769 (3)	0.7630 (3)	0.0110 (7)	
C34	0.3966 (3)	0.7386 (3)	0.8440 (3)	0.0131 (8)	
H34	0.3771	0.6968	0.8816	0.016*	
C35	0.4958 (2)	0.81260 (17)	0.92017 (18)	0.0152 (8)	
C36	0.5220 (2)	0.89622 (19)	0.89992 (17)	0.0242 (10)	
H36	0.4782	0.9082	0.8375	0.029*	
C37	0.6122 (3)	0.96228 (16)	0.9709 (2)	0.0311 (11)	
H37	0.6301	1.0194	0.9571	0.037*	
C38	0.6763 (2)	0.94472 (19)	1.0622 (2)	0.0322 (11)	
H38	0.7379	0.9899	1.1108	0.039*	
C39	0.6501 (2)	0.8611 (2)	1.08246 (17)	0.0322 (11)	
H39	0.6939	0.8491	1.1448	0.039*	
C40	0.5599 (2)	0.79503 (16)	1.0114 (2)	0.0253 (10)	
H40	0.5420	0.7379	1.0253	0.030*	
C41	0.29135 (18)	0.78216 (19)	0.79501 (19)	0.0138 (8)	
C42	0.2700 (2)	0.8122 (2)	0.7083 (2)	0.0235 (10)	
H42	0.3210	0.8049	0.6755	0.028*	
C43	0.1739 (2)	0.8529 (2)	0.66953 (19)	0.0290 (11)	
H43	0.1593	0.8734	0.6103	0.035*	
C44	0.09925 (19)	0.8636 (2)	0.7175 (2)	0.0267 (10)	
H44	0.0336	0.8914	0.6910	0.032*	
C45	0.1206 (2)	0.8335 (2)	0.8042 (2)	0.0235 (9)	
H45	0.0696	0.8408	0.8370	0.028*	
C46	0.2167 (2)	0.7928 (2)	0.84299 (17)	0.0196 (9)	
H46	0.2313	0.7723	0.9023	0.023*	
C47	0.5914 (3)	0.4068 (3)	0.7913 (3)	0.0128 (7)	
C48	0.6309 (3)	0.3932 (3)	0.8978 (3)	0.0147 (8)	
H48	0.5642	0.3636	0.9048	0.018*	
C49	0.72011 (19)	0.32657 (17)	0.9159 (2)	0.0163 (8)	
C50	0.8342 (2)	0.36122 (15)	0.9587 (2)	0.0242 (10)	
H50	0.8583	0.4271	0.9769	0.029*	
C51	0.91314 (17)	0.2995 (2)	0.9748 (2)	0.0306 (11)	
H51	0.9911	0.3231	1.0041	0.037*	
C52	0.8779 (2)	0.20305 (19)	0.9482 (2)	0.0296 (11)	
H52	0.9319	0.1608	0.9592	0.035*	
C53	0.7638 (3)	0.16840 (14)	0.9054 (2)	0.0282 (11)	
H53	0.7398	0.1025	0.8872	0.034*	
C54	0.68492 (18)	0.23016 (18)	0.8892 (2)	0.0226 (9)	
H54	0.6069	0.2065	0.8600	0.027*	
C55	0.6707 (2)	0.49094 (15)	0.97491 (17)	0.0147 (8)	
C56	0.6557 (2)	0.50229 (16)	1.06492 (19)	0.0197 (9)	
H56	0.6152	0.4527	1.0748	0.024*	
C57	0.7000 (2)	0.58622 (19)	1.14048 (16)	0.0238 (10)	
H57	0.6898	0.5940	1.2020	0.029*	
C58	0.7593 (2)	0.65881 (16)	1.12602 (18)	0.0233 (9)	
H58	0.7896	0.7162	1.1777	0.028*	
C59	0.7743 (2)	0.64746 (16)	1.0360 (2)	0.0241 (10)	
H59	0.8148	0.6971	1.0261	0.029*	
C60	0.7300 (2)	0.56353 (18)	0.96046 (16)	0.0214 (9)	
H60	0.7402	0.5558	0.8989	0.026*	

### Atomic displacement parameters (Å^2^)

**Table:**

	*U*^11^	*U*^22^	*U*^33^	*U*^12^	*U*^13^	*U*^23^
Sn1	0.01146 (13)	0.00818 (13)	0.00688 (13)	0.00179 (9)	0.00281 (10)	0.00295 (9)
Sn2	0.01089 (13)	0.00863 (13)	0.00698 (13)	0.00201 (9)	0.00306 (10)	0.00332 (10)
Sn3	0.01056 (13)	0.00959 (13)	0.00707 (13)	0.00261 (9)	0.00233 (10)	0.00380 (10)
O1	0.0171 (14)	0.0137 (14)	0.0097 (13)	0.0059 (11)	0.0033 (11)	0.0041 (11)
O2	0.0154 (13)	0.0118 (13)	0.0122 (14)	0.0021 (11)	0.0039 (11)	0.0039 (11)
O3	0.0164 (13)	0.0117 (13)	0.0096 (13)	0.0007 (10)	0.0063 (11)	0.0011 (10)
O4	0.0196 (14)	0.0097 (13)	0.0127 (14)	0.0023 (11)	0.0083 (12)	0.0026 (11)
O5	0.0135 (13)	0.0172 (14)	0.0094 (13)	0.0036 (11)	0.0027 (11)	0.0076 (11)
O6	0.0139 (13)	0.0185 (14)	0.0100 (13)	0.0042 (11)	0.0042 (11)	0.0085 (11)
O7	0.0117 (12)	0.0084 (12)	0.0089 (13)	0.0015 (10)	0.0038 (10)	0.0028 (10)
O8	0.0110 (12)	0.0101 (13)	0.0078 (12)	0.0018 (10)	0.0015 (10)	0.0045 (10)
O9	0.0126 (13)	0.0103 (13)	0.0080 (13)	0.0044 (10)	0.0037 (10)	0.0039 (10)
C1	0.0140 (18)	0.0089 (18)	0.0132 (19)	0.0000 (14)	0.0042 (15)	−0.0003 (15)
C2	0.018 (2)	0.020 (2)	0.024 (2)	0.0037 (16)	0.0083 (18)	0.0112 (18)
C3	0.021 (2)	0.018 (2)	0.047 (3)	0.0009 (17)	0.011 (2)	0.013 (2)
C4	0.013 (2)	0.027 (3)	0.036 (3)	−0.0017 (18)	0.001 (2)	0.001 (2)
C5	0.027 (3)	0.043 (3)	0.018 (2)	−0.007 (2)	0.000 (2)	0.001 (2)
C6	0.022 (2)	0.033 (3)	0.016 (2)	−0.0050 (19)	0.0012 (18)	0.0092 (19)
C7	0.0144 (18)	0.0158 (19)	0.0081 (18)	−0.0014 (15)	0.0024 (15)	0.0032 (15)
C8	0.016 (2)	0.025 (2)	0.023 (2)	0.0041 (17)	0.0072 (18)	0.0052 (18)
C9	0.014 (2)	0.041 (3)	0.026 (2)	0.0023 (19)	0.0073 (19)	0.004 (2)
C10	0.020 (2)	0.040 (3)	0.042 (3)	−0.010 (2)	0.013 (2)	−0.001 (2)
C11	0.032 (3)	0.018 (2)	0.047 (3)	−0.008 (2)	0.016 (2)	0.002 (2)
C12	0.019 (2)	0.019 (2)	0.028 (2)	−0.0007 (17)	0.0087 (19)	0.0024 (19)
C13	0.0104 (17)	0.0141 (19)	0.0092 (18)	−0.0002 (14)	0.0019 (15)	0.0001 (15)
C14	0.020 (2)	0.019 (2)	0.015 (2)	0.0026 (16)	0.0057 (17)	0.0055 (16)
C15	0.023 (2)	0.027 (2)	0.022 (2)	−0.0082 (19)	0.0044 (19)	0.0063 (19)
C16	0.016 (2)	0.030 (3)	0.029 (3)	−0.0046 (18)	0.0028 (19)	0.007 (2)
C17	0.019 (2)	0.031 (3)	0.023 (2)	0.0042 (18)	−0.0002 (19)	0.009 (2)
C18	0.016 (2)	0.021 (2)	0.018 (2)	0.0036 (16)	0.0019 (17)	0.0083 (17)
C19	0.0186 (19)	0.0099 (18)	0.0139 (19)	0.0043 (15)	0.0100 (16)	0.0057 (15)
C20	0.019 (2)	0.0084 (18)	0.0128 (19)	0.0036 (14)	0.0042 (16)	0.0045 (15)
C21	0.021 (2)	0.0121 (18)	0.017 (2)	0.0066 (15)	0.0079 (17)	0.0109 (16)
C22	0.022 (2)	0.020 (2)	0.019 (2)	0.0054 (17)	0.0058 (18)	0.0083 (17)
C23	0.022 (2)	0.030 (3)	0.025 (2)	0.0009 (19)	0.0019 (19)	0.013 (2)
C24	0.018 (2)	0.033 (3)	0.039 (3)	0.0088 (19)	0.012 (2)	0.023 (2)
C25	0.031 (2)	0.029 (2)	0.036 (3)	0.018 (2)	0.025 (2)	0.018 (2)
C26	0.028 (2)	0.022 (2)	0.018 (2)	0.0093 (18)	0.0118 (19)	0.0110 (18)
C27	0.021 (2)	0.0121 (19)	0.0077 (18)	0.0009 (15)	0.0019 (16)	0.0023 (15)
C28	0.024 (2)	0.020 (2)	0.019 (2)	0.0044 (17)	0.0095 (18)	0.0122 (17)
C29	0.025 (2)	0.025 (2)	0.025 (2)	0.0061 (18)	0.013 (2)	0.0077 (19)
C30	0.032 (2)	0.026 (2)	0.019 (2)	−0.0058 (19)	0.013 (2)	0.0078 (19)
C31	0.034 (3)	0.014 (2)	0.018 (2)	−0.0019 (18)	0.0069 (19)	0.0075 (17)
C32	0.027 (2)	0.0126 (19)	0.016 (2)	0.0045 (16)	0.0068 (18)	0.0035 (16)
C33	0.0087 (17)	0.0133 (18)	0.0081 (17)	0.0033 (14)	−0.0006 (14)	0.0039 (14)
C34	0.0191 (19)	0.0112 (18)	0.0121 (19)	0.0032 (15)	0.0090 (16)	0.0038 (15)
C35	0.021 (2)	0.0142 (19)	0.0115 (19)	0.0063 (16)	0.0082 (16)	0.0026 (15)
C36	0.030 (2)	0.023 (2)	0.019 (2)	−0.0032 (18)	0.0095 (19)	0.0062 (18)
C37	0.038 (3)	0.020 (2)	0.031 (3)	−0.007 (2)	0.016 (2)	−0.001 (2)
C38	0.026 (2)	0.026 (3)	0.030 (3)	−0.004 (2)	0.004 (2)	−0.006 (2)
C39	0.029 (3)	0.029 (3)	0.024 (3)	0.003 (2)	−0.002 (2)	0.000 (2)
C40	0.029 (2)	0.019 (2)	0.022 (2)	0.0017 (18)	0.004 (2)	0.0071 (18)
C41	0.0173 (19)	0.0116 (18)	0.0108 (19)	0.0031 (15)	0.0044 (16)	0.0017 (15)
C42	0.029 (2)	0.029 (2)	0.021 (2)	0.0147 (19)	0.0125 (19)	0.0161 (19)
C43	0.031 (3)	0.031 (3)	0.028 (3)	0.011 (2)	0.010 (2)	0.017 (2)
C44	0.020 (2)	0.022 (2)	0.034 (3)	0.0067 (18)	0.004 (2)	0.008 (2)
C45	0.015 (2)	0.023 (2)	0.031 (3)	−0.0003 (17)	0.0101 (19)	0.0030 (19)
C46	0.022 (2)	0.017 (2)	0.021 (2)	0.0020 (17)	0.0113 (18)	0.0029 (17)
C47	0.0169 (19)	0.0115 (18)	0.0088 (18)	0.0006 (14)	0.0034 (15)	0.0036 (14)
C48	0.0181 (19)	0.017 (2)	0.0113 (19)	0.0032 (15)	0.0059 (16)	0.0084 (16)
C49	0.020 (2)	0.022 (2)	0.0089 (18)	0.0070 (16)	0.0044 (16)	0.0078 (16)
C50	0.023 (2)	0.023 (2)	0.025 (2)	0.0062 (18)	0.0064 (19)	0.0068 (19)
C51	0.022 (2)	0.037 (3)	0.029 (3)	0.011 (2)	0.004 (2)	0.009 (2)
C52	0.036 (3)	0.037 (3)	0.022 (2)	0.022 (2)	0.012 (2)	0.015 (2)
C53	0.049 (3)	0.019 (2)	0.021 (2)	0.007 (2)	0.015 (2)	0.0088 (19)
C54	0.031 (2)	0.020 (2)	0.014 (2)	0.0013 (18)	0.0056 (18)	0.0063 (17)
C55	0.0140 (19)	0.018 (2)	0.0118 (19)	0.0049 (15)	0.0035 (15)	0.0062 (16)
C56	0.023 (2)	0.023 (2)	0.017 (2)	0.0051 (17)	0.0092 (18)	0.0099 (17)
C57	0.026 (2)	0.034 (3)	0.012 (2)	0.0097 (19)	0.0071 (18)	0.0056 (18)
C58	0.023 (2)	0.022 (2)	0.017 (2)	0.0037 (17)	0.0023 (18)	−0.0001 (18)
C59	0.025 (2)	0.022 (2)	0.023 (2)	−0.0017 (18)	0.0083 (19)	0.0046 (19)
C60	0.029 (2)	0.021 (2)	0.016 (2)	0.0020 (18)	0.0095 (18)	0.0069 (17)

### Geometric parameters (Å, °)

**Table:**

Sn1—O9^i^	2.070 (3)	C23—H23	0.9500
Sn1—O8	2.082 (3)	C24—C25	1.3900
Sn1—O7	2.094 (3)	C24—H24	0.9500
Sn1—O3	2.153 (3)	C25—C26	1.3900
Sn1—O1	2.170 (3)	C25—H25	0.9500
Sn1—C1	2.1336 (18)	C26—H26	0.9500
Sn2—O7	2.072 (3)	C27—C28	1.3900
Sn2—O9	2.088 (3)	C27—C32	1.3900
Sn2—O8	2.089 (3)	C28—C29	1.3900
Sn2—O4	2.163 (3)	C28—H28	0.9500
Sn2—O5	2.171 (3)	C29—C30	1.3900
Sn2—C7	2.1250 (18)	C29—H29	0.9500
Sn3—O7^i^	2.073 (3)	C30—C31	1.3900
Sn3—O8	2.076 (3)	C30—H30	0.9500
Sn3—O9	2.125 (3)	C31—C32	1.3900
Sn3—O2^i^	2.148 (3)	C31—H31	0.9500
Sn3—O6	2.158 (3)	C32—H32	0.9500
Sn3—C13	2.1181 (18)	C33—C34	1.523 (5)
O1—C19	1.267 (5)	C34—C35	1.531 (4)
O2—C19	1.255 (5)	C34—C41	1.535 (4)
O2—Sn3^i^	2.148 (3)	C34—H34	1.0000
O3—C33	1.252 (5)	C35—C36	1.3900
O4—C33	1.266 (4)	C35—C40	1.3900
O5—C47	1.261 (5)	C36—C37	1.3900
O6—C47	1.257 (5)	C36—H36	0.9500
O7—Sn3^i^	2.073 (3)	C37—C38	1.3900
O9—Sn1^i^	2.070 (3)	C37—H37	0.9500
C1—C2	1.3900	C38—C39	1.3900
C1—C6	1.3900	C38—H38	0.9500
C2—C3	1.3900	C39—C40	1.3900
C2—H2	0.9500	C39—H39	0.9500
C3—C4	1.3900	C40—H40	0.9500
C3—H3	0.9500	C41—C42	1.3900
C4—C5	1.3900	C41—C46	1.3900
C4—H4	0.9500	C42—C43	1.3900
C5—C6	1.3900	C42—H42	0.9500
C5—H5	0.9500	C43—C44	1.3900
C6—H6	0.9500	C43—H43	0.9500
C7—C8	1.3900	C44—C45	1.3900
C7—C12	1.3900	C44—H44	0.9500
C8—C9	1.3900	C45—C46	1.3900
C8—H8	0.9500	C45—H45	0.9500
C9—C10	1.3900	C46—H46	0.9500
C9—H9	0.9500	C47—C48	1.532 (5)
C10—C11	1.3900	C48—C49	1.538 (4)
C10—H10	0.9500	C48—C55	1.543 (4)
C11—C12	1.3900	C48—H48	1.0000
C11—H11	0.9500	C49—C50	1.3900
C12—H12	0.9500	C49—C54	1.3900
C13—C14	1.3900	C50—C51	1.3900
C13—C18	1.3900	C50—H50	0.9500
C14—C15	1.3900	C51—C52	1.3900
C14—H14	0.9500	C51—H51	0.9500
C15—C16	1.3900	C52—C53	1.3900
C15—H15	0.9500	C52—H52	0.9500
C16—C17	1.3900	C53—C54	1.3900
C16—H16	0.9500	C53—H53	0.9500
C17—C18	1.3900	C54—H54	0.9500
C17—H17	0.9500	C55—C56	1.3900
C18—H18	0.9500	C55—C60	1.3900
C19—C20	1.522 (5)	C56—C57	1.3900
C20—C21	1.524 (4)	C56—H56	0.9500
C20—C27	1.554 (4)	C57—C58	1.3900
C20—H20	1.0000	C57—H57	0.9500
C21—C22	1.3900	C58—C59	1.3900
C21—C26	1.3900	C58—H58	0.9500
C22—C23	1.3900	C59—C60	1.3900
C22—H22	0.9500	C59—H59	0.9500
C23—C24	1.3900	C60—H60	0.9500
			
O9^i^—Sn1—O8	104.81 (10)	C17—C18—H18	120.0
O9^i^—Sn1—O7	78.30 (10)	C13—C18—H18	120.0
O8—Sn1—O7	77.31 (10)	O2—C19—O1	125.2 (4)
O9^i^—Sn1—C1	110.39 (10)	O2—C19—C20	118.3 (3)
O8—Sn1—C1	98.50 (10)	O1—C19—C20	116.5 (3)
O7—Sn1—C1	171.19 (10)	C19—C20—C21	113.3 (3)
O9^i^—Sn1—O3	155.57 (10)	C19—C20—C27	109.1 (3)
O8—Sn1—O3	88.52 (10)	C21—C20—C27	112.9 (3)
O7—Sn1—O3	84.99 (10)	C19—C20—H20	107.1
C1—Sn1—O3	87.17 (10)	C21—C20—H20	107.1
O9^i^—Sn1—O1	84.06 (10)	C27—C20—H20	107.1
O8—Sn1—O1	163.51 (10)	C22—C21—C26	120.0
O7—Sn1—O1	91.25 (10)	C22—C21—C20	121.4 (2)
C1—Sn1—O1	91.14 (10)	C26—C21—C20	118.6 (2)
O3—Sn1—O1	78.56 (10)	C23—C22—C21	120.0
O9^i^—Sn1—Sn3^i^	40.85 (7)	C23—C22—H22	120.0
O8—Sn1—Sn3^i^	100.18 (7)	C21—C22—H22	120.0
O7—Sn1—Sn3^i^	39.51 (7)	C24—C23—C22	120.0
C1—Sn1—Sn3^i^	149.24 (7)	C24—C23—H23	120.0
O3—Sn1—Sn3^i^	117.45 (7)	C22—C23—H23	120.0
O1—Sn1—Sn3^i^	77.25 (7)	C23—C24—C25	120.0
O9^i^—Sn1—Sn2	100.89 (7)	C23—C24—H24	120.0
O8—Sn1—Sn2	39.88 (7)	C25—C24—H24	120.0
O7—Sn1—Sn2	39.46 (7)	C26—C25—C24	120.0
C1—Sn1—Sn2	134.14 (7)	C26—C25—H25	120.0
O3—Sn1—Sn2	76.13 (7)	C24—C25—H25	120.0
O1—Sn1—Sn2	125.52 (7)	C25—C26—C21	120.0
Sn3^i^—Sn1—Sn2	73.199 (8)	C25—C26—H26	120.0
O7—Sn2—O9	101.76 (10)	C21—C26—H26	120.0
O7—Sn2—O8	77.64 (10)	C28—C27—C32	120.0
O9—Sn2—O8	78.04 (10)	C28—C27—C20	123.3 (2)
O7—Sn2—C7	104.87 (10)	C32—C27—C20	116.6 (2)
O9—Sn2—C7	100.46 (10)	C29—C28—C27	120.0
O8—Sn2—C7	177.34 (10)	C29—C28—H28	120.0
O7—Sn2—O4	87.45 (10)	C27—C28—H28	120.0
O9—Sn2—O4	162.12 (10)	C28—C29—C30	120.0
O8—Sn2—O4	89.21 (10)	C28—C29—H29	120.0
C7—Sn2—O4	91.78 (10)	C30—C29—H29	120.0
O7—Sn2—O5	159.26 (10)	C31—C30—C29	120.0
O9—Sn2—O5	87.69 (10)	C31—C30—H30	120.0
O8—Sn2—O5	86.49 (10)	C29—C30—H30	120.0
C7—Sn2—O5	91.27 (10)	C32—C31—C30	120.0
O4—Sn2—O5	78.98 (10)	C32—C31—H31	120.0
O7^i^—Sn3—O8	104.46 (10)	C30—C31—H31	120.0
O7^i^—Sn3—C13	102.46 (10)	C31—C32—C27	120.0
O8—Sn3—C13	102.73 (10)	C31—C32—H32	120.0
O7^i^—Sn3—O9	77.53 (10)	C27—C32—H32	120.0
O8—Sn3—O9	77.49 (10)	O3—C33—O4	125.9 (4)
C13—Sn3—O9	179.77 (11)	O3—C33—C34	117.2 (3)
O7^i^—Sn3—O2^i^	88.73 (10)	O4—C33—C34	116.9 (3)
O8—Sn3—O2^i^	155.77 (10)	C33—C34—C35	111.4 (3)
C13—Sn3—O2^i^	93.90 (10)	C33—C34—C41	110.1 (3)
O9—Sn3—O2^i^	85.87 (10)	C35—C34—C41	112.7 (3)
O7^i^—Sn3—O6	160.60 (10)	C33—C34—H34	107.5
O8—Sn3—O6	85.71 (10)	C35—C34—H34	107.5
C13—Sn3—O6	91.05 (10)	C41—C34—H34	107.5
O9—Sn3—O6	88.91 (10)	C36—C35—C40	120.0
O2^i^—Sn3—O6	76.39 (10)	C36—C35—C34	121.4 (2)
O7^i^—Sn3—Sn1^i^	39.98 (7)	C40—C35—C34	118.6 (2)
O8—Sn3—Sn1^i^	100.15 (7)	C37—C36—C35	120.0
C13—Sn3—Sn1^i^	140.36 (7)	C37—C36—H36	120.0
O9—Sn3—Sn1^i^	39.57 (7)	C35—C36—H36	120.0
O2^i^—Sn3—Sn1^i^	76.81 (7)	C36—C37—C38	120.0
O6—Sn3—Sn1^i^	122.72 (7)	C36—C37—H37	120.0
O7^i^—Sn3—Sn2	99.93 (7)	C38—C37—H37	120.0
O8—Sn3—Sn2	39.57 (7)	C39—C38—C37	120.0
C13—Sn3—Sn2	140.41 (8)	C39—C38—H38	120.0
O9—Sn3—Sn2	39.79 (7)	C37—C38—H38	120.0
O2^i^—Sn3—Sn2	118.92 (7)	C40—C39—C38	120.0
O6—Sn3—Sn2	77.22 (7)	C40—C39—H39	120.0
Sn1^i^—Sn3—Sn2	73.224 (8)	C38—C39—H39	120.0
C19—O1—Sn1	128.6 (2)	C39—C40—C35	120.0
C19—O2—Sn3^i^	129.8 (3)	C39—C40—H40	120.0
C33—O3—Sn1	131.7 (2)	C35—C40—H40	120.0
C33—O4—Sn2	128.0 (2)	C42—C41—C46	120.0
C47—O5—Sn2	129.2 (2)	C42—C41—C34	123.3 (2)
C47—O6—Sn3	129.6 (3)	C46—C41—C34	116.7 (2)
Sn2—O7—Sn3^i^	134.38 (13)	C41—C42—C43	120.0
Sn2—O7—Sn1	100.58 (11)	C41—C42—H42	120.0
Sn3^i^—O7—Sn1	100.51 (11)	C43—C42—H42	120.0
Sn3—O8—Sn1	130.74 (13)	C42—C43—C44	120.0
Sn3—O8—Sn2	101.15 (11)	C42—C43—H43	120.0
Sn1—O8—Sn2	100.40 (11)	C44—C43—H43	120.0
Sn1^i^—O9—Sn2	134.17 (13)	C45—C44—C43	120.0
Sn1^i^—O9—Sn3	99.58 (10)	C45—C44—H44	120.0
Sn2—O9—Sn3	99.57 (11)	C43—C44—H44	120.0
C2—C1—C6	120.0	C44—C45—C46	120.0
C2—C1—Sn1	123.29 (14)	C44—C45—H45	120.0
C6—C1—Sn1	115.69 (13)	C46—C45—H45	120.0
C1—C2—C3	120.0	C45—C46—C41	120.0
C1—C2—H2	120.0	C45—C46—H46	120.0
C3—C2—H2	120.0	C41—C46—H46	120.0
C4—C3—C2	120.0	O6—C47—O5	126.3 (4)
C4—C3—H3	120.0	O6—C47—C48	116.8 (3)
C2—C3—H3	120.0	O5—C47—C48	116.9 (3)
C3—C4—C5	120.0	C47—C48—C49	112.2 (3)
C3—C4—H4	120.0	C47—C48—C55	108.7 (3)
C5—C4—H4	120.0	C49—C48—C55	113.2 (3)
C4—C5—C6	120.0	C47—C48—H48	107.5
C4—C5—H5	120.0	C49—C48—H48	107.5
C6—C5—H5	120.0	C55—C48—H48	107.5
C5—C6—C1	120.0	C50—C49—C54	120.0
C5—C6—H6	120.0	C50—C49—C48	121.2 (2)
C1—C6—H6	120.0	C54—C49—C48	118.8 (2)
C8—C7—C12	120.0	C49—C50—C51	120.0
C8—C7—Sn2	121.10 (14)	C49—C50—H50	120.0
C12—C7—Sn2	118.76 (13)	C51—C50—H50	120.0
C7—C8—C9	120.0	C52—C51—C50	120.0
C7—C8—H8	120.0	C52—C51—H51	120.0
C9—C8—H8	120.0	C50—C51—H51	120.0
C8—C9—C10	120.0	C51—C52—C53	120.0
C8—C9—H9	120.0	C51—C52—H52	120.0
C10—C9—H9	120.0	C53—C52—H52	120.0
C9—C10—C11	120.0	C54—C53—C52	120.0
C9—C10—H10	120.0	C54—C53—H53	120.0
C11—C10—H10	120.0	C52—C53—H53	120.0
C12—C11—C10	120.0	C53—C54—C49	120.0
C12—C11—H11	120.0	C53—C54—H54	120.0
C10—C11—H11	120.0	C49—C54—H54	120.0
C11—C12—C7	120.0	C56—C55—C60	120.0
C11—C12—H12	120.0	C56—C55—C48	118.2 (2)
C7—C12—H12	120.0	C60—C55—C48	121.5 (2)
C14—C13—C18	120.0	C55—C56—C57	120.0
C14—C13—Sn3	120.39 (13)	C55—C56—H56	120.0
C18—C13—Sn3	119.59 (13)	C57—C56—H56	120.0
C13—C14—C15	120.0	C58—C57—C56	120.0
C13—C14—H14	120.0	C58—C57—H57	120.0
C15—C14—H14	120.0	C56—C57—H57	120.0
C16—C15—C14	120.0	C57—C58—C59	120.0
C16—C15—H15	120.0	C57—C58—H58	120.0
C14—C15—H15	120.0	C59—C58—H58	120.0
C15—C16—C17	120.0	C60—C59—C58	120.0
C15—C16—H16	120.0	C60—C59—H59	120.0
C17—C16—H16	120.0	C58—C59—H59	120.0
C18—C17—C16	120.0	C59—C60—C55	120.0
C18—C17—H17	120.0	C59—C60—H60	120.0
C16—C17—H17	120.0	C55—C60—H60	120.0
C17—C18—C13	120.0		
			
O9^i^—Sn1—O1—C19	38.1 (3)	C9—C10—C11—C12	0.0
O8—Sn1—O1—C19	−85.5 (5)	C10—C11—C12—C7	0.0
O7—Sn1—O1—C19	−40.0 (3)	C8—C7—C12—C11	0.0
C1—Sn1—O1—C19	148.5 (3)	Sn2—C7—C12—C11	175.7 (2)
O3—Sn1—O1—C19	−124.6 (3)	O7^i^—Sn3—C13—C14	48.36 (17)
Sn3^i^—Sn1—O1—C19	−2.8 (3)	O8—Sn3—C13—C14	−59.83 (17)
Sn2—Sn1—O1—C19	−61.0 (3)	O2^i^—Sn3—C13—C14	137.91 (16)
O9^i^—Sn1—O3—C33	85.9 (4)	O6—Sn3—C13—C14	−145.66 (16)
O8—Sn1—O3—C33	−38.3 (3)	Sn1^i^—Sn3—C13—C14	63.94 (19)
O7—Sn1—O3—C33	39.1 (3)	Sn2—Sn3—C13—C14	−74.62 (18)
C1—Sn1—O3—C33	−136.9 (3)	O7^i^—Sn3—C13—C18	−130.11 (16)
O1—Sn1—O3—C33	131.4 (3)	O8—Sn3—C13—C18	121.71 (16)
O7—Sn2—O4—C33	−35.7 (3)	O2^i^—Sn3—C13—C18	−40.55 (16)
O9—Sn2—O4—C33	86.0 (4)	O6—Sn3—C13—C18	35.87 (16)
O8—Sn2—O4—C33	41.9 (3)	Sn1^i^—Sn3—C13—C18	−114.53 (14)
C7—Sn2—O4—C33	−140.5 (3)	Sn2—Sn3—C13—C18	106.92 (15)
O5—Sn2—O4—C33	128.5 (3)	C18—C13—C14—C15	0.0
O7—Sn2—O5—C47	−70.5 (4)	Sn3—C13—C14—C15	−178.5 (2)
O9—Sn2—O5—C47	47.5 (3)	C13—C14—C15—C16	0.0
O8—Sn2—O5—C47	−30.7 (3)	C14—C15—C16—C17	0.0
C7—Sn2—O5—C47	147.9 (3)	C15—C16—C17—C18	0.0
O4—Sn2—O5—C47	−120.5 (3)	C16—C17—C18—C13	0.0
O7^i^—Sn3—O6—C47	−86.2 (4)	C14—C13—C18—C17	0.0
O8—Sn3—O6—C47	36.5 (3)	Sn3—C13—C18—C17	178.47 (19)
C13—Sn3—O6—C47	139.2 (3)	Sn3^i^—O2—C19—O1	−21.4 (6)
O9—Sn3—O6—C47	−41.0 (3)	Sn3^i^—O2—C19—C20	156.3 (3)
O2^i^—Sn3—O6—C47	−127.0 (3)	Sn1—O1—C19—O2	14.0 (6)
O9—Sn2—O7—Sn3^i^	26.1 (2)	Sn1—O1—C19—C20	−163.7 (2)
O8—Sn2—O7—Sn3^i^	100.93 (18)	O2—C19—C20—C21	41.0 (5)
C7—Sn2—O7—Sn3^i^	−78.16 (18)	O1—C19—C20—C21	−141.1 (3)
O4—Sn2—O7—Sn3^i^	−169.33 (18)	O2—C19—C20—C27	−85.6 (4)
O5—Sn2—O7—Sn3^i^	141.8 (2)	O1—C19—C20—C27	92.2 (4)
O9—Sn2—O7—Sn1	−90.12 (11)	C19—C20—C21—C22	−63.7 (4)
O8—Sn2—O7—Sn1	−15.33 (10)	C27—C20—C21—C22	60.9 (3)
C7—Sn2—O7—Sn1	165.57 (10)	C19—C20—C21—C26	116.3 (3)
O4—Sn2—O7—Sn1	74.41 (11)	C27—C20—C21—C26	−119.0 (3)
O5—Sn2—O7—Sn1	25.6 (3)	C26—C21—C22—C23	0.0
O9^i^—Sn1—O7—Sn2	123.71 (12)	C20—C21—C22—C23	−179.9 (3)
O8—Sn1—O7—Sn2	15.40 (10)	C21—C22—C23—C24	0.0
O3—Sn1—O7—Sn2	−74.21 (11)	C22—C23—C24—C25	0.0
O1—Sn1—O7—Sn2	−152.62 (11)	C23—C24—C25—C26	0.0
O9^i^—Sn1—O7—Sn3^i^	−15.61 (10)	C24—C25—C26—C21	0.0
O8—Sn1—O7—Sn3^i^	−123.92 (12)	C22—C21—C26—C25	0.0
O3—Sn1—O7—Sn3^i^	146.47 (11)	C20—C21—C26—C25	179.9 (3)
O1—Sn1—O7—Sn3^i^	68.07 (11)	C19—C20—C27—C28	−11.7 (4)
O7^i^—Sn3—O8—Sn1	−26.24 (19)	C21—C20—C27—C28	−138.6 (2)
C13—Sn3—O8—Sn1	80.43 (17)	C19—C20—C27—C32	171.7 (2)
O9—Sn3—O8—Sn1	−99.64 (17)	C21—C20—C27—C32	44.8 (3)
O2^i^—Sn3—O8—Sn1	−147.4 (2)	C32—C27—C28—C29	0.0
O6—Sn3—O8—Sn1	170.53 (17)	C20—C27—C28—C29	−176.5 (3)
O7^i^—Sn3—O8—Sn2	88.14 (11)	C27—C28—C29—C30	0.0
C13—Sn3—O8—Sn2	−165.20 (10)	C28—C29—C30—C31	0.0
O9—Sn3—O8—Sn2	14.74 (10)	C29—C30—C31—C32	0.0
O2^i^—Sn3—O8—Sn2	−33.0 (3)	C30—C31—C32—C27	0.0
O6—Sn3—O8—Sn2	−75.09 (11)	C28—C27—C32—C31	0.0
O9^i^—Sn1—O8—Sn3	25.36 (19)	C20—C27—C32—C31	176.8 (3)
O7—Sn1—O8—Sn3	99.42 (17)	Sn1—O3—C33—O4	2.6 (6)
C1—Sn1—O8—Sn3	−88.47 (17)	Sn1—O3—C33—C34	−176.8 (2)
O3—Sn1—O8—Sn3	−175.38 (17)	Sn2—O4—C33—O3	−4.8 (5)
O1—Sn1—O8—Sn3	146.4 (3)	Sn2—O4—C33—C34	174.6 (2)
O9^i^—Sn1—O8—Sn2	−89.33 (11)	O3—C33—C34—C35	−46.6 (4)
O7—Sn1—O8—Sn2	−15.27 (10)	O4—C33—C34—C35	133.9 (3)
C1—Sn1—O8—Sn2	156.85 (11)	O3—C33—C34—C41	79.2 (4)
O3—Sn1—O8—Sn2	69.93 (11)	O4—C33—C34—C41	−100.3 (4)
O1—Sn1—O8—Sn2	31.7 (4)	C33—C34—C35—C36	81.7 (3)
O7—Sn2—O8—Sn3	−120.03 (12)	C41—C34—C35—C36	−42.7 (4)
O9—Sn2—O8—Sn3	−14.98 (10)	C33—C34—C35—C40	−98.8 (3)
O4—Sn2—O8—Sn3	152.40 (11)	C41—C34—C35—C40	136.9 (2)
O5—Sn2—O8—Sn3	73.40 (11)	C40—C35—C36—C37	0.0
O7—Sn2—O8—Sn1	15.41 (10)	C34—C35—C36—C37	179.6 (3)
O9—Sn2—O8—Sn1	120.47 (12)	C35—C36—C37—C38	0.0
O4—Sn2—O8—Sn1	−72.15 (11)	C36—C37—C38—C39	0.0
O5—Sn2—O8—Sn1	−151.16 (12)	C37—C38—C39—C40	0.0
O7—Sn2—O9—Sn1^i^	−24.5 (2)	C38—C39—C40—C35	0.0
O8—Sn2—O9—Sn1^i^	−98.97 (18)	C36—C35—C40—C39	0.0
C7—Sn2—O9—Sn1^i^	83.26 (18)	C34—C35—C40—C39	−179.6 (3)
O4—Sn2—O9—Sn1^i^	−144.3 (3)	C33—C34—C41—C42	−36.9 (4)
O5—Sn2—O9—Sn1^i^	174.13 (18)	C35—C34—C41—C42	88.1 (3)
O7—Sn2—O9—Sn3	89.02 (11)	C33—C34—C41—C46	144.5 (3)
O8—Sn2—O9—Sn3	14.54 (10)	C35—C34—C41—C46	−90.5 (3)
C7—Sn2—O9—Sn3	−163.23 (10)	C46—C41—C42—C43	0.0
O4—Sn2—O9—Sn3	−30.8 (4)	C34—C41—C42—C43	−178.6 (3)
O5—Sn2—O9—Sn3	−72.36 (11)	C41—C42—C43—C44	0.0
O7^i^—Sn3—O9—Sn1^i^	15.37 (10)	C42—C43—C44—C45	0.0
O8—Sn3—O9—Sn1^i^	123.49 (11)	C43—C44—C45—C46	0.0
O2^i^—Sn3—O9—Sn1^i^	−74.24 (11)	C44—C45—C46—C41	0.0
O6—Sn3—O9—Sn1^i^	−150.67 (11)	C42—C41—C46—C45	0.0
O7^i^—Sn3—O9—Sn2	−122.79 (11)	C34—C41—C46—C45	178.7 (3)
O8—Sn3—O9—Sn2	−14.67 (10)	Sn3—O6—C47—O5	9.6 (6)
O2^i^—Sn3—O9—Sn2	147.60 (11)	Sn3—O6—C47—C48	−168.4 (2)
O6—Sn3—O9—Sn2	71.17 (11)	Sn2—O5—C47—O6	−13.1 (6)
Sn1^i^—Sn3—O9—Sn2	−138.16 (15)	Sn2—O5—C47—C48	165.0 (2)
O9^i^—Sn1—C1—C2	38.31 (19)	O6—C47—C48—C49	−29.8 (5)
O8—Sn1—C1—C2	147.65 (17)	O5—C47—C48—C49	151.9 (3)
O3—Sn1—C1—C2	−124.27 (17)	O6—C47—C48—C55	96.2 (4)
O1—Sn1—C1—C2	−45.78 (17)	O5—C47—C48—C55	−82.1 (4)
O9^i^—Sn1—C1—C6	−153.31 (15)	C47—C48—C49—C50	93.4 (3)
O8—Sn1—C1—C6	−43.97 (16)	C55—C48—C49—C50	−30.1 (4)
O3—Sn1—C1—C6	44.12 (15)	C47—C48—C49—C54	−87.0 (3)
O1—Sn1—C1—C6	122.61 (15)	C55—C48—C49—C54	149.5 (2)
C6—C1—C2—C3	0.0	C54—C49—C50—C51	0.0
Sn1—C1—C2—C3	167.9 (2)	C48—C49—C50—C51	179.6 (3)
C1—C2—C3—C4	0.0	C49—C50—C51—C52	0.0
C2—C3—C4—C5	0.0	C50—C51—C52—C53	0.0
C3—C4—C5—C6	0.0	C51—C52—C53—C54	0.0
C4—C5—C6—C1	0.0	C52—C53—C54—C49	0.0
C2—C1—C6—C5	0.0	C50—C49—C54—C53	0.0
Sn1—C1—C6—C5	−168.79 (19)	C48—C49—C54—C53	−179.6 (3)
O7—Sn2—C7—C8	−46.94 (18)	C47—C48—C55—C56	147.7 (2)
O9—Sn2—C7—C8	−152.21 (16)	C49—C48—C55—C56	−87.0 (3)
O4—Sn2—C7—C8	40.90 (17)	C47—C48—C55—C60	−38.7 (4)
O5—Sn2—C7—C8	119.91 (17)	C49—C48—C55—C60	86.7 (3)
O7—Sn2—C7—C12	137.38 (16)	C60—C55—C56—C57	0.0
O9—Sn2—C7—C12	32.10 (17)	C48—C55—C56—C57	173.8 (3)
O4—Sn2—C7—C12	−134.79 (17)	C55—C56—C57—C58	0.0
O5—Sn2—C7—C12	−55.78 (17)	C56—C57—C58—C59	0.0
C12—C7—C8—C9	0.0	C57—C58—C59—C60	0.0
Sn2—C7—C8—C9	−175.6 (2)	C58—C59—C60—C55	0.0
C7—C8—C9—C10	0.0	C56—C55—C60—C59	0.0
C8—C9—C10—C11	0.0	C48—C55—C60—C59	−173.5 (3)

Symmetry codes: (i) −*x*+1, −*y*+1, −*z*+1.
